# Supplementary material for: Alterations in lipid profiles in children with perinatally acquired HIV infection living in Ghana: A cross-sectional study
Source: PLoS One. 2025 Jul 17;20(7):e0318314. doi: 10.1371/journal.pone.0318314 (PMC12270137; doi:10.1371/journal.pone.0318314)
Supplement: S1 Table — (DOCX) [file pone.0318314.s001.docx]

**Alterations in lipid profiles in children with perinatally acquired HIV infection living in Ghana: a cross-sectional study**

Ruth Ayanful-Torgby^1,2^*, Veronika Shabanova^1,3^, Akosua Aya Essuman^2^, Emmanuel Boafo^2^, Linda Eva Amoah^2^, Elijah Paintsil^1,4*^

**S1 Table** Comparison of dyslipidemia prevalence by sex

| **Dyslipidemia, n (%)** | **Female (n=202)** | **Male (n=195)** | **p-value^1^** |
| --- | --- | --- | --- |
| TC | 25 (12.38) | 22 (11.28) | 0.49 |
| TG | 32 (15.84) | 31(15.90) | 0.50 |
| LDL-C | 23 (11.39) | 13 (6.67) | 0.13 |
| HDL-C | 49 (24.25) | 47(24.10) | 0.50 |
| Total proportion | 83 (43.34) | 85 (43.34) | 0.61 |

^1^Chi-square test or Fisher’s Exact Test. Abbreviations: low density lipoprotein (LDL-C), high triglycerides (TG), cholesterol (TC), and low high-density lipoprotein (HDL-C)

**S2 Table** Comparison of lipid profiles by sex

| **Parameter, mean (SD)** | **Female (n=202)** | **Male (n=195)** | **p-value^i^** |
| --- | --- | --- | --- |
| TC (mg/dL) | 157.42 (47.30) | 154.66 (42.73) | 0.54 |
| TG (mg/dL) | 97.61 (55.30) | 96.13 (56.91) | 0.79 |
| LDL-C (mg/dL) | 69.50 (57.48) | 64.38 (45.69) | 0.34 |
| HDL-C mg/dL) | 43.56 (14.39) | 46.60 (17.41) | 0.43 |

^i^ Student’s t-test. Abbreviations: low density lipoprotein (LDL-C), high triglycerides (TG), cholesterol (TC), and low high-density lipoprotein (HDL-C)
